# The Impact of Marijuana Legalization on Vehicular Trauma

**DOI:** 10.7759/cureus.3671

**Published:** 2018-12-03

**Authors:** Natash Keric, Luke J Hofmann, Rachelle Babbitt-Jonas, Joel Michalek, Mathew Dolich, Leen Khoury, Javier Martin Perez, Stephen M Cohn

**Affiliations:** 1 Surgery, Banner University Medical, Phoenix, USA; 2 Surgery, University of Texas Health Science Center, San Antonio, USA; 3 Epidemiology and Biostatistics, University of Texas at San Antonio, San Antonio, USA; 4 Surgery, University of California, Orange, USA; 5 Surgery, Staten Island University Hospital, Staten Island, USA; 6 Surgery, Hackensack Meridian Health, Hackensack, USA

**Keywords:** marijuana, alcohol intoxication, drugged driving, motor vehicle collisions, trauma injury, marijuana legalization

## Abstract

Background

Drugged driving, or driving under the influence of any drug, is a growing public health concern, especially with the recent legislation legalizing marijuana use in certain states in the USA. We sought to gain a better understanding of the surgeons’ perspective regarding marijuana (MJ) and alcohol (ETOH) and the relationship of recent laws to identification of MJ and ETOH in trauma victims.

Methods

Members of a national trauma surgical organization were asked to participate in an Institutional Review Board (IRB)-approved, web-based survey which centered on attitudes, knowledge, and beliefs regarding ETOH and MJ as they related to injury. Two Level I trauma center registries (located in TX and CA) were queried for the incidence of motor vehicular collision (MVC) and the presence of ETOH (defined as > 0.08 g/dL) or MJ from 2006 thru 2012.

Results

A total of 127 trauma surgeons participated in the survey. The majority were male (84%, n = 107) and with a median age of 52. Most were in surgical practice for greater than 11 years (78%, n = 99) and worked at a Level I trauma center (78%, n = 99) in an academic institution (65%, n = 83). MJ was illegal in the states where most of the participants were in practice (79%, n = 100), but 90% (n = 114) of respondents from states where MJ is legal stated they have not seen an increase in MVC since MJ was legalized. At the TX trauma center, only 4% of patients involved in a vehicular trauma tested positive for MJ, 21% of patients had the presence of ETOH, and 3% had both. For both MJ and also ETOH, the incidence remained the same each year. In CA, there was little yearly variation in the incidence of patients that tested positive for MJ (23%), ETOH (50%), and both (7%). In addition, the incidence of MJ was essentially unchanged after the decriminalization law was passed in 2010.

Conclusion

The prevalence of cannabis and alcohol varies among the states studied, TX and CA. The impact of decriminalization of marijuana did not seem to affect the incidence of drugged driving with marijuana in CA.

## Introduction

Drugged driving, or driving under the influence of any drug, is a growing public health concern, especially with the recent legislation legalizing marijuana use in certain states in the USA. As of 2018, 31 states and the District of Columbia have legalized marijuana for medicinal use [1]. According to the most recent National Survey on Drug Use and Health, marijuana is by far the most commonly used illicit drug [2]. In fact, 18.9 million Americans over the age of 12 used cannabis during 2012 [2] and 10.3 million persons admit to driving under the influence of illicit drugs.

Overall, marijuana is the most prevalent illegal drug detected in impaired drivers, fatally injured drivers, and motor vehicle crash victims [3]. The relationship between marijuana use and motor vehicle crashes has been previously studied from both experimental and observational approaches. Simulator and laboratory studies have found that the active component of marijuana, 9-tetrahydrocannabinol (THC), is associated with decreased driving performance [4]. It has been established that acutely intoxicated marijuana users show impairment on cognitive, perceptual, and psychomotor tasks [5]. Marijuana has also been shown to slow reaction time, affect decision-making, and increase risk-taking behavior [6, 7]. Optimal cognitive skills are essential when operating a motor vehicle and any compromise is a public health concern.

In 2000, the National Highway Traffic Safety Administration (NHTSA), developed a drug information sheet based on an international panel of experts on drug impaired driving. The panel’s assessment of driving risks while under the influence of marijuana reported that even low doses moderately impair cognitive and psychomotor tasks; whereas high doses, chronic use, and use in combination with low doses of alcohol, severely impair driving [8]. Despite this knowledge, drugged driving laws are lagging behind alcohol impaired driving legislation, owing to a lack of consensus on the drug intoxication limit and determination of current drug levels among the different states. With new data to support an increase in drivers who test positive for illicit drugs, there is an impetus to understand how drugs are affecting our road traffic safety [3].

Trauma surgeons, by virtue of their profession, have a unique perspective regarding drivers under the influence. At the crux of this field is injury prevention. Knowledge of the current policies and literature allows the trauma surgeon to gain a greater appreciation of how injuries affect our healthcare system. We sought to gain a better understanding of the trauma surgeons’ perspective regarding marijuana (MJ) and alcohol (ETOH), and also determine the actual relationship of recent laws to the findings of MJ and ETOH in trauma victims.

## Materials and methods

After obtaining institutional review board approval from the University of Texas Health Science Center at San Antonio (UTHSCSA), an email invitation to participate in an electronic survey was sent to members of the American Association for the Surgery of Trauma (AAST) in July 2013. The email invitation included a brief description and purpose of the survey with an electronic link to access the online site, Survey MonkeyÒ. Upon entering the website, members were asked to voluntarily and anonymously agree or decline participation in the survey. There was no identifying information collected on the Survey MonkeyÒ site. The survey was open only for 30 days. The email explained the purpose of the study, a confidentiality statement, and a link to the survey should they choose to participate. The participants were asked a series of questions, which centered on attitudes, knowledge, and beliefs regarding alcohol and marijuana as they related to traumatic injury. Descriptive statistics were reported for the demographic characteristics. Questionnaire responses were tabulated and reported as frequency (%).

Trauma center registries at The University of California Irvine (UCI) and UTHSCSA were queried for the incidence of motor vehicular collision (MVC) and the presence of ETOH (defined as >0.08 g/dL) or MJ (defined as >50 ng/ml in TX and >100 ng/ml in CA) or both from 2006 thru 2012. The impact of decriminalization in CA in 2010 on drugged diving was assessed during this time frame.

## Results

A total of 127 trauma surgeons participated and completed the survey. The majority were male (84%, n = 107) and in the Baby Boomers age group, 49–66 (56%, n = 71). Generation X (29–48) and the Silent Generation (67–88) made up 37% (n = 47) and 7% (n = 9) of respondents, respectively. The median age was 51.5 years with a standard deviation of 9.5. Twenty-two percent of respondents (n = 28) have been in surgical practice for less than a decade, 39% (n = 49) have been in practice for less than 20 years, and 39% (n = 50) have been in practice for more than 20 years. The majority (78%, n = 99) reported working at a Level I trauma center, 17% (n = 22) at a Level II trauma center, and less than 2% (n = 3) either in a Level III trauma center or a community hospital. Most surgeons worked at an academic institution (65%, n = 83), with other locations being community institutions (20%, n = 25), private institutions (9%, n = 11), and military institutions (3%, n = 4). Current location of practice was varied in survey respondents, with the most represented region being the Southern United States (33%, n = 42), followed by Western (24%, n = 31), Midwestern (18%, n = 23), and the Northeastern United States (16%, n = 20). The majority of the respondents had not served in the military (75%, n = 95).

Seventy-nine percent of the AAST survey respondents (n = 100) answered that they practice in a state that had not legalized or decriminalized marijuana. Most of the participants that answered this question actually were from Non-Legal States (NLS), while 17% (n = 22) answered incorrectly and were in fact practicing in Legal States (LS). Less than 2% (n = 3) of participants believed that there was an increase in MVCs since marijuana legalization. The majority (43%, n = 55) believed that 0–20% of their patients involved in MVCs tested positive for marijuana, followed by 21–40%. When surveyed on what percentage of drivers involved in MVCs tested for marijuana and another substance, the majority answered that they did not know, followed by 21–40% of their patients. Seventy-five percent of respondents (n = 95) agreed that marijuana use does affect one’s ability to drive and 67% (n = 85) believed alcohol-related MVCs are the most common trauma seen in the emergency department.

A total of 24,926 patients were seen and treated at UTHSCSA, a Level I trauma center, between 2006 and 2012. Twenty-nine percent (7,171) were involved in either a motor vehicle collision or a motorcycle collision (MCC). All patients were screened for alcohol and drug abuse. Twenty-one percent of post collision patients tested positive for alcohol. With regard to marijuana, 4% of collision patients were found to be positive for marijuana. Only 3% of patients involved in a vehicular trauma screened positive for both marijuana and alcohol (Table 1).

**Table 1:** UTHSCSA vehicular trauma with a positive ethanol, positive marijuana, or positive both ethanol and marijuana blood test. UTHSCSA: University of Texas Health Science Center at San Antonio

Year	Total trauma	Vehicular trauma (VT)	Positive ethanol (%)	Positive marijuana (%)	Positive ethanol and marijuana (%)
2006	3165	1010	179 (17)	30 (3)	22 (2)
2007	3551	995	209 (21)	24 (2)	39 (4)
2008	3619	1024	213 (21)	44 (4)	36 (3)
2009	3375	921	221 (24)	41 (4)	38 (4)
2010	3357	971	224 (23)	47 (5)	33 (3)
2011	3641	1077	228 (21)	34 (3)	28 (3)
2012	4218	1173	221( 19)	39 (3)	26 (2)
TOTALS	24926	7171	1495 (21)	259 (4)	222 (3)

Similar data was collected at UCI, a comparable Level I trauma center in California where a total of 16,084 trauma victims were involved in a vehicular MVC during the same time period. Of these patients, 50% tested positive for alcohol, 23% tested positive for marijuana and 7% tested positive for both substances. There was a higher percentage of patients who were under the influence of marijuana at UCI (despite the fact that a positive test was defined by the UCI laboratory as >100 ng/ml, compared to 50 ng/ml at UTHSCSA); however, there did not appear to be an increase in the percentage of patients who tested positive after decriminalization of the drug went into effect in 2010 (Table 2).

**Table 2:** UCI vehicular trauma with positive ethanol, positive marijuana, or positive both ethanol and marijuana blood test. UCI: University of California Irvine

Year	Total trauma	Vehicular trauma (VT)	Positive ethanol (%)	Positive marijuana (%)	Positive ethanol and marijuana (%)
2006	1799	903	429 (48)	188 (20)	58 (6)
2007	1941	954	491 (51)	192 (20)	62 (6)
2008	1983	971	489 (50)	179 (18)	48 (5)
2009	2117	790	466 (59)	185 (23)	40 (5)
2010	2220	910	484 (53)	220 (24)	67 (7)
2011	2801	1268	598 (47)	285 (22)	82 (6)
2012	3223	1524	711 (47)	399 (26)	123 (8)
TOTALS	16084	7320	3668 (50)	1648 (23)	480 (7)

## Discussion

Impaired driving is a huge problem in the United States. Driving under the influence of alcohol increases the risk of both non-fatal and fatal collisions [9]. Motor vehicle crashes account for approximately 33,000 deaths per year, with 32% of total traffic fatalities related to alcohol-impaired driving [10]. As of 2011, all 50 states, the District of Columbia, and Puerto Rico have adopted the per se law making it illegal to drive with a blood alcohol content (BAC) of 0.08 g/dl or higher [11]. In fatal crashes in 2011, drivers with a BAC of 0.08 was highest for drivers ages 21–24 (32%), followed by ages 25–34 (30%) and 35–44 (24%) [2]. Interestingly, drivers involved in fatal crashes with a BAC of 0.08 or higher were seven times more likely to have had prior driving while impaired (DWI) convictions [11]. Alcohol-impaired motor vehicle crashes cost more than an estimated $37 billion annually [11]. While the past 10 years has been characterized by a decline in alcohol-impaired driving by 27%, it is still a significant cause of morbidity and mortality here in the United States [11].

In 2011, 10.3 million, or 4% of the population aged 12 or older reported driving under the influence of illicit drugs [5]. The rate of driving under the influence of illicit drugs was highest among young adults aged 18 to 25, but had the highest rate of increase in those 26 and older [5]. Nationwide, in 2009, 63% of fatally injured drivers were tested for drug involvement, and 18% were positive [6].

Currently, there are 31 states that have legalized medical marijuana use and decriminalized cannabis. There is a significant public health concern that the legalization of marijuana has created an opportunity scenario conducive to increased drugged driving. Ongoing debates from both sides of the political fence are divided on the issue, centering on the repercussions and benefits of this legislation. Most of the participants in our survey felt that the use of marijuana affects one’s driving ability. This belief is echoed in a recent systematic review and meta-analysis which concluded that acute consumption of marijuana doubles the risk of a motor vehicle collision resulting in serious injury or death [12]. However, other studies have concluded that total traffic fatalities have decreased in the states that have legalized marijuana, mostly due to marijuana being used as a substitute for alcohol and that users consume it more frequently at home rather than driving to public locations [13].

This area of contention might explain why drugged driving laws are lagging behind drunk driving laws. Many studies cite the considerable variability and inconsistency of the data in discerning dose and time dependent impairment in the field and implementing laboratory tests in practice [12]. There are also misconceptions that driving under the influence of drugs is not as serious of a threat as driving drunk. Even with simulator studies that show impairment on cognitive, perceptual and psychomotor tasks while drugged driving, the levels of impairment do not match laboratory values and in one study there were no effects of marijuana on driving after 24 hours of administration [14, 15].

In our own study, we found different practice patterns between the two institutions evaluated. At UTHSCSA, the cutoff for detection of THC in urine was 50 ng/ml, whereas, at UCI, the cutoff was 100 ng/ml. Because of the lack of standardized drugged driving laws, there is a paucity of data that can accurately estimate the prevalence of marijuana use among drivers and the impact on vehicular trauma and public health. Clearly, there is a need for continued research to ascertain to what degree marijuana affects driving performance and risk of vehicular trauma.

To date, there has been no study that evaluates whether there has been an increase in MVC secondary to drugged driving in states that have cannabis decriminalization. We sought to answer this question and queried data from The University of California-Irvine, located in a recently decriminalized state and at UTHSCSA, located in a non-legal state. Our survey showed that there was no change in the percentage of drugged drivers involved in vehicular trauma in either institution during the period studied. UCI had a higher percentage of drivers driving under the influence of marijuana with a higher concentration cutoff for detection of the drug in the urine when compared to UTHSCSA. Therefore, there may have been potentially more drugged drivers reported if a lower cutoff was used at UCI. The percentage does not appear to correlate to the California decriminalization act in 2010, however, the institution had much higher percentage of drugged drivers compared to those evaluated in a non-legal state institution, UTHSCSA. Trauma surgeons queried in our survey grossly overestimated the prevalence of marijuana use amongst drivers involved in collisions. The majority believed that up to 40% of their trauma patients test positive for marijuana. Interestingly, more respondents from states where MJ is legal believed the incidence to be lower at 0–20% than those from states where it remains illegal, who thought it was higher at 21–40%. Data from UTHSCSA revealed just 4% of patients involved in an MVC tested positive for marijuana. When comparing the data at UCI, the number is much higher at 23%, but is still significantly less than what the majority of the respondents believed. The estimation by the respondents seems to also exceed national data which showed that among daytime drivers, 11% tested positive for drugs, compared to 14% of nighttime drivers [16]. These results are similar to the study done by The European Monitoring Centre for Drugs and Drug Addiction which showed that across 14 studies conducted in Australia, Denmark, France, the Netherlands, and the United States between 1993 and 2005, the rates of cannabis detected (primarily via blood samples) in drivers injured in traffic crashes ranged from 3.3% to 26.9% (11.8% on average). Furthermore, the review revealed that among the 23 studies of drivers killed in car crashes, cannabis was detected in 1.4% to 37% of drivers (11.7% on average) [17].

Twenty-five years ago, “Have one [drink] for the road” was a commonly used phrase in American culture. According to the NHTSA Traffic Safety 2011 facts, however, the number of fatal crashes involving alcohol impaired driving has decreased in the past 10 years [11]. This decline has been attributed to the concerted efforts of many organizations and public awareness involved in influencing legislation.

Our survey found that 67% (n = 85) of trauma surgeons responding believe that alcohol-related MVCs are the most common trauma seen in the emergency department. National data report that 31% of total motor vehicle traffic fatalities are alcohol-related, a decrease of 2.5% from the previous year. When looking at our own data at UTHSCSA, 21% of vehicular trauma victims tested positive for alcohol, and the percentage stayed relatively stable throughout the time period studied. At UCI, the percentage of vehicular drivers who tested positive for alcohol was double that of UTHSCSA. Again, the incidence seems to have remained stable throughout the time period studied. Our data reflects national data which shows that the incidence of alcohol amongst vehicular trauma has plateaued and is on the decline. Unlike testing for THC, BAC testing is standardized, which allows for more accurate comparisons between institutions and will likely serve as a model for national recognition and education of the effects that drug driving has on MVC.

Laboratory studies have shown that use of marijuana and alcohol is synergistic, and the combined effects are typically greater than the effects of marijuana alone. In our study, 21% (n = 27) of respondents believed that 21–40% of their patients that test positive for marijuana also test positive for another drug (ethanol, cocaine, opioid). When looking at the data from UTHSCSA, only 3% of the trauma victims tested positive for alcohol and marijuana over the time period studied. The number reported at UCI was higher at 7%. In both studies, the number stayed relatively stable across all the years, even with marijuana decriminalization in California. A large Australian study by Drummer et al. found that of 3,400 fatally injured drivers, 10% of the cases involved both alcohol and drugs [18]. In their meta-analysis of experimental studies regarding the effects of marijuana dose on driving performance, Grotenhermen et al. identified that a THC concentration of 7–10 ng/ml in blood serum would result in a comparable risk of crash to a blood alcohol content of 0.05% [19]. There has been no large study done in the US that has looked at the incidence of alcohol and marijuana use on vehicular trauma.

## Conclusions

The prevalence of cannabis and alcohol varies among the states studied, TX and CA. The impact of decriminalization of marijuana did not seem to affect the incidence of drugged driving with marijuana in CA.
